# Rehabilitative Impact of Exercise Training on Human Skeletal Muscle Transcriptional Programs in Parkinson’s Disease

**DOI:** 10.3389/fphys.2020.00653

**Published:** 2020-06-17

**Authors:** Kaleen M. Lavin, Yongchao Ge, Stuart C. Sealfon, Venugopalan D. Nair, Katarzyna Wilk, Jeremy S. McAdam, Samuel T. Windham, Preeti Lakshman Kumar, Merry-Lynn N. McDonald, Marcas M. Bamman

**Affiliations:** ^1^Department of Cell, Developmental and Integrative Biology, School of Medicine, University of Alabama at Birmingham, Birmingham, AL, United States; ^2^UAB Center for Exercise Medicine, School of Medicine, University of Alabama at Birmingham, Birmingham, AL, United States; ^3^Department of Neurology, Icahn School of Medicine at Mount Sinai, New York, NY, United States; ^4^Center for Advanced Research on Diagnostic Assays, Icahn School of Medicine at Mount Sinai, New York, NY, United States; ^5^Department of Surgery, School of Medicine, University of Alabama at Birmingham, Birmingham, AL, United States; ^6^Department of Genetics, School of Medicine, University of Alabama at Birmingham, Birmingham, AL, United States; ^7^Department of Medicine, School of Medicine, University of Alabama at Birmingham, Birmingham, AL, United States; ^8^Birmingham/Atlanta VA Geriatric Research, Education, and Clinical Center, Birmingham, AL, United States; ^9^Department of Neurology, School of Medicine, University of Alabama at Birmingham, Birmingham, AL, United States

**Keywords:** Parkinson’s disease, high-intensity exercise training, transcriptome, neuromuscular, motor unit

## Abstract

Parkinson’s disease (PD) is the most common motor neurodegenerative disease, and neuromuscular function deficits associated with PD contribute to disability. Targeting these symptoms, our laboratory has previously evaluated 16-week high-intensity resistance exercise as rehabilitative training (RT) in individuals with PD. We reported significant improvements in muscle mass, neuromuscular function (strength, power, and motor unit activation), indices of neuromuscular junction integrity, total and motor scores on the unified Parkinson’s disease rating scale (UPDRS), and total and sub-scores on the 39-item PD Quality of Life Questionnaire (PDQ-39), supporting the use of RT to reverse symptoms. Our objective was to identify transcriptional networks that may contribute to RT-induced neuromuscular remodeling in PD. We generated transcriptome-wide skeletal muscle RNA-sequencing in 5 participants with PD [4M/1F, 67 ± 2 years, Hoehn and Yahr stages 2 (*n* = 3) and 3 (*n* = 2)] before and after 16-week high intensity RT to identify transcriptional networks that may in part underpin RT-induced neuromuscular remodeling in PD. Following RT, 304 genes were significantly upregulated, notably related to remodeling and nervous system/muscle development. Additionally, 402 genes, primarily negative regulators of muscle adaptation, were downregulated. We applied the recently developed Pathway-Level Information ExtractoR (PLIER) method to reveal coordinated gene programs (as latent variables, LVs) that differed in skeletal muscle among young (YA) and old (OA) healthy adults and PD (*n* = 12 per cohort) at baseline and in PD pre- vs. post-RT. Notably, one LV associated with angiogenesis, axon guidance, and muscle remodeling was significantly lower in PD than YA at baseline and was significantly increased by exercise. A different LV annotated to denervation, autophagy, and apoptosis was increased in both PD and OA relative to YA and was also reduced by 16-week RT in PD. Thus, this analysis identified two novel skeletal muscle transcriptional programs that are dysregulated by PD and aging, respectively. Notably, RT has a normalizing effect on both programs in individuals with PD. These results identify potential molecular transducers of the RT-induced improvements in neuromuscular remodeling and motor function that may aid in optimizing exercise rehabilitation strategies for individuals with PD.

## Introduction

Parkinson’s disease (PD) is a common neurological disorder affecting approximately one in 100 adults over age 65. While its root cause stemming from death of dopaminergic neurons in the *substantia nigra* is well-established, the functionally disruptive impact of PD on muscle function is poorly understood. In addition to its roles in locomotion and gait, skeletal muscle is an active participant in tissue cross-talk via secretion of signaling factors produced in muscle tissue (e.g., myokines), which may impact neural function and health (Delezie and Handschin, 2018; Pedersen, 2019). Fittingly, muscular loading through exercise training has received attention as a neuroprotective (Alkadhi, 2018) or even neurorestorative (Azizi and Vendrame, 2007) therapy, with wide-ranging benefits for neuromuscular function and cognitive health in PD (Ferreira et al., 2018; Marusiak et al., 2019; Tollar et al., 2019).

Our laboratory has demonstrated that 16 weeks of high-intensity resistance exercise rehabilitation training (RT) not only restores skeletal muscle mass and strength to levels found in healthy adults but also leads to improvements in cognition, well-being, and both overall and motor-specific domains of the Unified Parkinson’s Disease Rating Score (UPDRS) and 39-item Parkinson’s Disease Questionnaire (PDQ-39), validated metrics of PD severity (Kelly et al., 2014). Furthermore, fMRI analysis performed immediately after a single acute bout of this RT regimen demonstrates heightened activity in key brain regions, including the *substantia nigra* and prefrontal cortex (Kelly et al., 2017). If these acute effects are predictive of motor and cognitive adaptations to training (Voss et al., 2019) this may provide a mechanistic link to improvements in both motor and non-motor symptoms of PD seen following 16-week RT. It is likely that exercise training elicits an integrated physiological response that leads to these improvements and that key insight may be gained by examining skeletal muscle, which may play a communicative role in these beneficial adaptations.

Our laboratory has also previously shown that coordinated motor unit activation in individuals with PD markedly improves following RT, indicating reduced difficulty performing a functional sit-to-stand task (Kelly et al., 2014). This improvement is believed to be a reflection of an RT-induced reduction in average motor unit size, which has previously been shown to increase with aging (Kelly et al., 2018b; Roberts et al., 2018) and specifically PD (Caviness et al., 2002; Kelly et al., 2018a; Lavin et al., 2019). The histological manifestation of motor unit remodeling in the *vastus lateralis* is type I myofiber grouping, a skeletal muscle phenotype noticeable in aged adults (Stalberg and Fawcett, 1982; Piasecki et al., 2016) but further exaggerated in individuals with PD (Kelly et al., 2018a). Notably, 16-week RT partially reduces the degree of type I grouping in a manner consistent with improvements in motor unit activation and PD-specific indices of disease progression. Thus, along with modulating the severity of other symptoms, exercise-induced demands placed on skeletal muscle partially undo a hallmark pathology in peripheral tissue. We have previously investigated this phenotype extensively (Kelly et al., 2018a, b; Lavin et al., 2019) demonstrating that muscle communication with the nervous system is concordantly altered. Due to the apparently active role of muscle in signaling to promote survival, reinnervation, and remodeling, maintenance of muscle health through exercise may be of critical importance in PD or older individuals at risk for PD. While exercise dose-response trials are needed to explore the intensity threshold for these effects, our data suggest high-intensity RT has profound effects on motor unit remodeling, likely because all motor units are recruited during near-maximal to maximal contractions (unlike steady-state endurance exercise).

We hypothesized that RT-induced improvements in PD pathology, some of which were fully recovered to healthy levels, would be reflected in the skeletal muscle transcriptome of individuals with PD following RT. To interrogate this, we generated transcriptome-wide skeletal muscle gene expression in a subset of individuals with PD before and after the training regimen detailed in previous work by our laboratory (Kelly et al., 2014). Data were analyzed using a recently developed bioinformatics framework entitled Pathway-Level Information ExtractoR (PLIER) (Mao et al., 2019) to identify molecular programs impacted by 16-week high intensity exercise training in persons with PD and to compare changes in gene expression to healthy old and young comparators. A more comprehensive understanding of the transcriptional profile associated with PD and reversed by exercise aids in illuminating potential mechanisms of neuromuscular remodeling and symptom improvement and identifying potential targets that could be exploited in future interventions leveraging combinatorial therapy.

## Materials and Methods

### Human Subjects

Five subjects with idiopathic PD, a subset of 15 participants in our previous RT trial (Kelly et al., 2014) provided skeletal muscle tissue samples for this study. All persons were Hoehn and Yahr stages 2 (*n* = 3) or 3 (*n* = 2) and medication stable for at least 4 weeks. For an additional exploratory analysis, we included seven additional baseline PD subjects, 12 sex-matched young adults (YA), and 12 age- and sex-matched healthy older adults (OA). Together with the five exercisers, these 36 individuals were previously extensively profiled at baseline in a transcriptome-wide RNA-Seq analysis of type I myofiber grouping (Lavin et al., 2019). Studies for which the individuals volunteered have been reported previously (Kosek et al., 2006; Merritt et al., 2013; Kelly et al., 2014) and detailed recruitment and eligibility information can be found therein. Both OA and YA were non-exercising, disease-free controls. All volunteers provided written informed consent to have their samples stored and utilized in future studies. Each study was reviewed and approved by the University of Alabama at Birmingham Institutional Review Board and conducted in accordance with the Declaration of Helsinki.

### High-Intensity Resistance Rehabilitative Training Intervention and Testing

The 16-week RT regimen was performed as previously reported in detail (Kelly et al., 2014, 2018a). Briefly, sessions (3 days/week) averaged 35–45 min (inter-subject variability due to differences in heart rate response, perceived fatigue, and degree of bradykinesia) and consisted of a combination of strength, power, endurance, balance, and functional training. Between sets of exercises targeting the large muscle groups (three sets of 8–12 repetitions to volitional fatigue for leg press, knee extension, chest press, overhead press, and lat pull down), participants performed functional mobility exercises (e.g., bodyweight squat, push-up, step-up, lunge, side lunge, modified dip for 45–60 s, or a 60 s interval on a treadmill or stationary cycle). Heart rate was maintained above 50% heart rate reserve (HRR) at all times and averaged ≥60% HRR during each session. Throughout the 16-week period, progression was incorporated as previously described (Kosek et al., 2006; Bamman et al., 2007). Briefly, resistance loads were increased when a subject completed 12 repetitions for two of three sets at a given resistance while maintaining proper form. Subjects also completed three sets of abdominal crunches each session. Skeletal muscle functional tests were performed as previously outlined in detail in order to assess the maximum load that could be successfully lifted one time (one-repetition maximum, 1RM) and the peak power achieved at a resistance of 45% of 1RM during knee extension exercise (Petrella et al., 2005; Kelly et al., 2014). Motor unit activation was assessed during a three-repetition functional sit-to-stand task and is represented as a percentage relative to maximum voluntary contraction measured via surface electromyography, as previously described (Petrella et al., 2005, 2007).

### Skeletal Muscle Biopsy and Immunohistochemistry

Skeletal muscle samples were obtained from the vastus lateralis under local anesthesia using a 5 mm Bergstrom biopsy needle with suction and processed to remove excess fat, blood, and connective tissue. Muscle samples to be used for RNA-Seq were snap-frozen in liquid nitrogen (LN_2_) and stored at −80°C until use. A portion of the muscle to be used for immunohistochemistry was mounted in tragacanth gum mixed with Tissue Tek O.C.T. compound (Sakura Finetek, Torrance, CA, United States) atop a square of cork, frozen in isopentane cooled to the temperature of LN_2_, and stored at −80°C.

Myofiber distribution, size, and grouping were determined from 6 μm skeletal muscle sections cut on a cryostat at −20°C, stained with antibodies against the myosin heavy chain I and IIa, imaged on a fluorescence microscope (BX51, Olympus, Tokyo, Japan), and analyzed using ImagePro Premier v9.1, as described in detail previously (Kelly et al., 2018b). All skeletal muscle histological and performance measures were compared between pre- and post-RT using a paired *t*-test or, if non-normally distributed, a Wilcoxon test in R, version 3.4.3 (R Core Team^1^).

### RNA Isolation and cDNA Library Synthesis

Skeletal muscle samples from the five PD subjects pre- and post-RT, along with 7 basal PD, 12 basal OA, and 12 basal YA (total *n* = 41, mean ± SD 7.5 ± 2.1 mg) were homogenized in a Bead Ruptor Elite bead mill homogenizer (Omni International, Kennesaw, GA, United States) at a speed of 4.2 m/s for 2 × 20 s while cooled by liquid nitrogen to 10°C. Muscle homogenates were processed using the Agencourt RNAdvance Tissue Kit (Beckman Coulter, Indianapolis, IN, United States) on a BioMek FX^P^ Laboratory Automation Workstation (Beckman Coulter, Indianapolis, IN, United States). The quality (260/280: 1.9 ± 0.0, 260/230: 1.4 ± 0.1) and integrity (RIN: 8.95 ± 0.05, 28S/18S: 1.6 ± 0.2) of isolated RNA (125 ± 44 ng/μL) were assessed using NanoDrop and an RNA Standard Sensitivity Kit (DNF-471, Advanced Analytical Technologies, Ankeny, IA, United States) on a Fragment Analyzer Automated CE system (Advanced Analytical Technologies). Subsequently, cDNA libraries were constructed from 250 ng of total RNA using the Universal Plus mRNA-Seq kit (NuGEN Technologies, San Carlos, CA, United States). Library concentration (75.8 ± 17.9 ng/μL) was assessed fluorometrically using the Qubit dsDNA HS Kit (Thermo Fisher), and quality (average fragment size: 336 ± 5 bp) was assessed with the Genomic DNA 50Kb Analysis Kit (DNF-467, Advanced Analytical Technologies).

### RNA Sequencing and Pre-processing

Preliminary sequencing of cDNA libraries (average read depth of 90 thousand reads) was performed using a MiSeq system (Illumina, Inc., San Diego, CA, United States) to confirm library quality. Deep sequencing was subsequently performed using an S2 flow cell in a NovaSeq sequencing system (Illumina) (average read depth = 25.5 million pairs of 2 × 50 bp reads). Raw data were processed using bcl2fastq Conversion Software (Illumina) to obtain FASTQ files, and the FASTQ files were aligned to the Human GENCODE hg38 genome using STAR (Dobin et al., 2013). Gene expression was quantified with featureCounts (Liao et al., 2014). The raw FASTQ data and the final gene expression matrix were deposited to GEO (accession number GSE140089).

### Gene Level Differential Expression Analysis

After filtering for low expression, RNA-Seq data were normalized using a voom correction and compared within the five PD subjects pre- vs. post-RT intervention. Differential expression analysis was performed using Bioconductor (Gentleman et al., 2004) package limma (Ritchie et al., 2015) under R version 3.4.3 (R Core Team^1^). Benjamini–Hochberg correction (Benjamini and Hochberg, 1995) for multiple comparisons testing was applied, setting the false discovery rate (FDR) < 0.05. Using R package biomaRt (Smedley et al., 2015) transcript Ensembl IDs were converted to gene names, when available, in order to allow for further annotation. Interpretation was limited to genes that exceeded a log2fold-change cutoff of 1 in either direction (i.e., expression doubled or halved following 16-week RT). While use of any stringent cutoff for fold-change is arbitrary, as a meaningful fold-change likely varies based on the biology of a given transcript, the decision to implement this threshold was influenced by our assessment of a large number of dimensions in a small group of individuals. Genes that surpassed the rigorous cutoff of ±1 log2FC were viewed as more robust findings that might hold up in future investigations with larger sample sizes.

### Pathway-Level Information ExtractoR

In order to identify transcriptional programs that were rescued by RT, a separate, complementary analysis using the PLIER framework (Mao et al., 2019) was performed on gene expression data obtained from all 41 skeletal muscle samples (12 basal PD, 5 post-training PD, 12 basal OA, and 12 basal YA). Count data were filtered for expression, retaining 17,786 transcripts, and log_2_ normalization for library size was applied across samples. 5,884 genes were retained in PLIER’s deconvolution algorithm. The underlying premise and mathematical approach have been previously described (Mao et al., 2019). Briefly, latent variables (LVs) represent eigengene-like patterns in gene expression across samples, where each LV serves as a single measurement to summarize a group of genes that tend to show similar regulatory changes. For each gene in an LV, the degree to which it contributes to the LV is computed as its “loading” (a column of matrix Z, Mao et al., 2019). The gene expression of each sample can be approximated by a linear combination of the LVs, and the coefficients of these linear combinations represent the LV “score” of a given sample (a row of matrix B, Mao et al., 2019). A key distinction of PLIER is the use of optimized decomposition of the gene expression in each sample into LVs guided by biological pathway gene sets to better capture the biological processes in the data. This is in contrast to a deconvolution method such as principal components analysis (PCA), which generates orthogonal principal components and does not utilize any prior biological knowledge. In contrast to PCA, PLIER LVs are not necessarily orthogonal to each other, and LV scores cannot be obtained by simple projection, but must be computed simultaneously with loadings via PLIER’s optimization procedure (Mao et al., 2019).

Presently, PLIER identified 25 latent variables (LV), and pathway designations were assigned based on prior knowledge. Each LV was assigned a number for further processing, and further descriptions of identified LVs are available in Table 4. For each LV, differences across the four groups were compared using a one-way ANOVA for the three basal groups (Basal OA vs. Basal YA vs. Basal PD) and a paired t-test for the five individuals with PD that underwent RT. Significant difference was declared at *P* < 0.05. LVs that were different at baseline in PD vs. either healthy group (OA or YA) as well as different between PD pre- to post-RT were investigated further. Within these LVs of interest, the top transcripts associated with the LV were manually annotated. When possible, preferential focus was given to their roles in human skeletal muscle or exercise adaptations; for many genes, data from other tissues or animal models constituted most of the available knowledge base.

## Results

Subject characteristics are presented in Table 1. The impact of the RT intervention has been previously published for a larger cohort (*n* = 15) (Kelly et al., 2014). For the current subset of five individuals, the RT intervention increased cross-sectional area of IIa fibers (+23%, *P* < 0.05) and trended strongly toward an expected IIa to IIx/IIax shift in fiber type distribution (IIa: +18%, *P* = 0.08; IIx/IIax: −13%, *P* = 0.06). Furthermore, RT improved the PDQ-39 total score (−8 points, *P* < 0.05) and led to sizable improvements in whole muscle strength and power (67%, *P* < 0.05 for each).

**TABLE 1:** Study subject characteristics.

	PD Pre-RT	PD Post-RT	% or point change
**Characteristics at baseline**			
Subjects (*n*)	5 (4M, 1F)		
Age (y)	67 ± 2		
Time since diagnosis (y)	5 ± 2		
Hoehn and Yahr stage	2 (*n* = 3),		
	3 (*n* = 2)		
Levodopa equivalency dose	455 ± 181		
**Parkinson’s disease progression**			
MDS-UPDRS total score	53 ± 7	47 ± 5	−6
Part I: Behavior/mentation/mood	11 ± 4	9 ± 3	−2
Part II: ADL	11 ± 3	11 ± 3	0
Part III: motor	29 ± 4	27 ± 4	−2
Part IV: dyskinesia	2 ± 1	1 ± 1	−1
PDQ-39 total score	38 ± 12	30 ± 11	−8*
ADL subscore	21 ± 3	12 ± 4	−9*
Mobility subscore	18 ± 8	17 ± 8	−1
Emotional well-being subscore	31 ± 8	21 ± 10	−10^†^
Cognitive impairment subscore	34 ± 9	23 ± 8	−11^†^
**Myofiber CSA (μm^2^)**			
Type I	4347 ± 342	5135 ± 399	21 ± 13
Type IIa	4532 ± 811	5582 ± 780	30 ± 15
Type IIx/IIax**	3762 ± 438	–	–
Type II total	3946 ± 514	5582 ± 780	42 ± 12*
**Myofiber distribution (%)**			
Type I	51 ± 4	46 ± 8	5 ± 6
Type IIa	34 ± 9	52 ± 8	18 ± 8^†^
Type IIx/IIax	15 ± 6	2 ± 1	−13 ± 5^†^
**Neuromuscular function**			
Knee extension 1RM, kg	77 ± 10	127 ± 14	67 ± 11*
Knee extension peak power, W	234 ± 44	364 ± 42	67 ± 18*
Motor unit activation (%)	0.68 ± 0.12	0.72 ± 0.16	5 ± 14
**Type I myofiber grouping**			
Mean group size	241 ± 151	142 ± 72	−18 ± 62
Percent of I grouped	57 ± 15	51 ± 18	−6 ± 17

Values are mean ± SE. PD, Parkinson’s disease; RT, 16 weeks of high intensity resistance exercise rehab training; MDS-UPDRS, Movement Disorder Society Unified Parkinson’s Disease Rating Scale; PDQ-39, 39-item Parkinson’s Disease Questionnaire; ADL, activities of daily living; CSA, cross-sectional area; 1RM, one repetition maximum; W, watts. **Too few IIx/IIax fibers (i.e., ≤25) were present at post-RT time point to obtain a representative myofiber CSA. *P < 0.05 vs. pre-RT; ^†^P < 0.10 vs. pre-RT. Data from the full study cohort are presented in a previous publication by our group (Kelly et al., 2014).

### Genes Differentially Expressed After 16-Week RT in PD Skeletal Muscle

**FIGURE 1:**
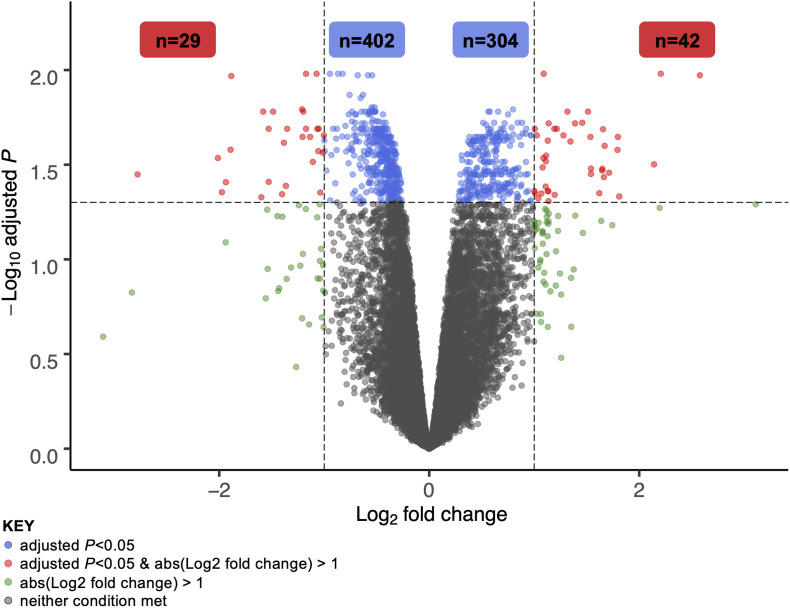
Volcano plot of differentially expressed genes in skeletal muscle of individuals with Parkinson’s disease following 16 weeks of high intensity resistance exercise rehabilitation training. A total of 706 genes were significantly different at the 16-week time point vs. pre-training (adjusted *P*-value, or FDR, <0.05). Of 304 total upregulated genes, 42 met or exceeded a log_2_ fold-change cutoff of 1 (i.e., expression doubled or more following RT). Of 402 total downregulated genes, 29 fell below a log_2_ fold-change cutoff of –1 (i.e., expression halved or less following RT). These gene sets are explored in greater detail in Tables 2, 3.

A total of 706 genes were differentially expressed (FDR < 0.05) in skeletal muscle of individuals with PD following 16-week RT (Figure 1). Of these, 304 genes were significantly upregulated (Supplementary Data Sheet 1). Transcripts meeting or exceeding a fold-change of 2.0 (*n* = 42) are shown in Table 2. Predominant biological functions in the upregulated gene set included muscle remodeling, muscle and nerve development, inflammation, and muscle metabolism. An additional 402 genes were downregulated following RT (Supplementary Data Sheet 2). Those at or below fold-change of 0.5 (*n* = 31) are shown in Table 3. Processes associated with downregulated genes included regulation of skeletal muscle growth, autophagy, and slow muscle metabolism.

**TABLE 2:** Skeletal muscle genes upregulated with RT in Parkinson’s disease.

Gene name	Description	Fold-change	FDR
**Skeletal muscle development and remodeling**			
IGFN1	Immunoglobulin-like and fibronectin type III domain containing 1	5.97	0.01
COL1A1	Collagen type I alpha 1 chain	3.15	0.02
COL3A1	Collagen type III alpha 1 chain	2.85	0.02
COL4A1	Collagen type IV alpha 1 chain	2.75	0.02
PANX1	Pannexin 1	2.54	0.02
CAPN6	Calpain 6	2.49	0.02
PXDN	Peroxidasin	2.13	0.01
AGPAT4	1-acylglycerol-3-phosphate *O*-acyltransferase 4	2.05	0.05
GJA1	Gap junction protein alpha 1	2.01	0.02
**Nervous system development**			
PCDH17	Protocadherin 17	3.51	0.05
HTR7	5-hydroxytryptamine receptor 7	3.18	0.03
ST8SIA2	ST8 alpha-*N*-acetyl-neuraminide alpha-2,8-sialyltransferase 2	3.13	0.03
ADCYAP1R1	ADCYAP receptor type I	2.32	0.02
FOXS1	forkhead box S1	2.19	0.04
HECW2	HECT, C2 and WW domain containing E3 ubiquitin protein ligase 2	2.19	0.02
NRP2	Neuropilin 2	2.12	0.03
**Inflammation and immunity**			
CXCL9	C-X-C motif chemokine ligand 9	3.47	0.03
FCN3	Ficolin 3	3.07	0.04
MXRA5	Matrix remodeling associated 5	2.91	0.03
COL4A2	Collagen type IV alpha 2 chain	2.43	0.02
NOS2	Nitric oxide synthase 2	2.29	0.05
FSCN1	Fascin actin-bundling protein 1	2.20	0.02
KCNC4	Potassium voltage-gated channel subfamily C member 4	2.16	0.04
LYZ	Lysozyme	2.10	0.04
**Skeletal muscle metabolism**			
GAL3ST3	Galactose-3-*O*-sulfotransferase 3	3.17	0.04
NR5A2	Nuclear receptor subfamily 5 group A member 2	2.91	0.04
EXOC3L1	Exocyst complex component 3 like 1	2.90	0.02
MTFP1	Mitochondrial fission process 1	2.28	0.02
SLC7A1	Solute carrier family 7 member 1	2.16	0.03
RNF152	Ring finger protein 152	2.04	0.02
**PD pathology**			
SIPA1L2	Signal induced proliferation associated 1 like 2	4.61	0.01
LOXHD1	Lipoxygenase homology domains 1	4.41	0.03
SCT	Secretin	3.47	0.02
VIPR1	Vasoactive intestinal peptide receptor 1	2.62	0.02
**Lesser known function**			
HGC6.3	Uncharacterized LOC100128124	3.28	0.03
AC087289.5	Novel transcript, antisense to TRIM47	3.14	0.03
ITPRIPL1	ITPRIP like 1	2.20	0.05
DSTNP2	Destrin, actin depolymerizing factor pseudogene 2	2.19	0.04
PITPNM1	Phosphatidylinositol transfer protein membrane associated 1	2.16	0.03
AC015878.1	Novel transcript, antisense to GREB1L	2.13	0.03
PCAT19	Prostate cancer associated transcript 19	2.01	0.04
C2CD4C	C2 calcium dependent domain containing 4C	2.00	0.04

*Fold-change in gene expression post-RT from pre-RT; FDR, false discovery rate.*

**TABLE 3:** Skeletal muscle genes downregulated with RT in Parkinson’s disease.

Gene name	Description	Fold-change	FDR
**Regulation of muscle growth and development**			
GREM2	Gremlin 2, DAN family BMP antagonist	0.15	0.04
NPTX1	Neuronal pentraxin 1	0.27	0.03
MSTN	Myostatin	0.33	0.02
ACTN3	Actinin alpha 3 (gene/pseudogene)	0.36	0.02
IL32	Interleukin 32	0.43	0.02
LOXL4	Lysyl oxidase like 4	0.44	0.02
RRAD	RRAD, Ras related glycolysis inhibitor and calcium channel regulator	0.46	0.02
TSPAN8	Tetraspanin 8	0.48	0.03
**Autophagy and apoptosis**			
NME9	NME/NM23 family member 9	0.35	0.02
ARG2	Arginase 2	0.35	0.04
GGT7	Gamma-glutamyltransferase 7	0.43	0.02
SH3RF2	SH3 domain containing ring finger 2	0.46	0.03
**Metabolism**			
GDA	Guanine deaminase	0.25	0.04
MYH1	Myosin heavy chain 1	0.26	0.04
CALML6	Calmodulin like 6	0.27	0.01
TRARG1	Trafficking regulator of GLUT4 (SLC2A4) 1	0.33	0.05
CACNA1E	Calcium voltage-gated channel subunit alpha1 E	0.38	0.05
PRKAG3	Protein kinase AMP-activated non-catalytic subunit gamma 3	0.44	0.01
SLC38A4	Solute carrier family 38 member 4	0.44	0.02
MYLK2	Myosin light chain kinase 2	0.48	0.01
GOLGA7B	Golgin A7 family member B	0.48	0.02
PARP15	Poly(ADP-ribose) polymerase family member 15	0.49	0.04
TTC39B	Tetratricopeptide repeat domain 39B	0.50	0.03
**Lesser known function**			
UNQ6494	Novel transcript	0.25	0.03
CFAP61	Cilia and flagella associated protein 61	0.38	0.02
LRRC3B	Leucine rich repeat containing 3B	0.39	0.04
FAM184B	Family with sequence similarity 184 member B	0.39	0.02
AC113133.1	Novel transcript, antisense to ANK1	0.48	0.02
ANKRD33B	Ankyrin repeat domain 33B	0.50	0.02

*Fold-change in gene expression post-RT from pre-RT; FDR, false discovery rate.*

**TABLE 4:** Latent variables (LVs) constructed by PLIER.

	Basal comparison			RT intervention		
	YA	OA	PD	PD pre-RT	PD post-RT	Annotation (if available)
LV1^‡^	0.49 ± 0.71	0.13 ± 1.22	−0.24 ± 0.62	−0.20 ± 0.28	−0.89 ± 0.41	REACTOME_GLUCOSE_METABOLISM
LV2^‡^	0.10 ± 0.94	−0.75 ± 1.09	0.07 ± 0.73	0.32 ± 0.65	1.40 ± 0.50	REACTOME_TCA_CYCLE_AND_RESPIRATORY_ELECTRON_ TRANSPORT
LV3^‡^	0.39 ± 0.87	−0.29 ± 1.12	−0.28 ± 0.57	−0.27 ± 0.59	0.41 ± 0.70	KEGG_PROTEASOME
LV4*^‡^	0.30 ± 0.67	−0.15 ± 0.61	−0.50 ± 0.55	−0.54 ± 0.33	0.84 ± 0.37	N/A
LV5	0.32 ± 0.60	−0.12 ± 0.69	−0.14 ± 0.89	−0.16 ± 0.8	−0.15 ± 0.84	MIPS_RIBOSOME_CYTOPLASMIC
LV6^†^	−0.48 ± 0.39	−0.15 ± 0.59	0.46 ± 0.67	0.70 ± 0.31	0.41 ± 0.59	KEGG_VALINE_LEUCINE_AND_ISOLEUCINE_DEGRADATION
LV7	−0.03 ± 0.71	−0.04 ± 0.76	0.21 ± 0.83	0.35 ± 0.75	−0.32 ± 0.34	REACTOME_NUCLEAR_RECEPTOR_TRANSCRIPTION_ PATHWAY
LV8	−0.32 ± 0.88	0.20 ± 0.79	0.11 ± 0.58	0.15 ± 0.52	0.02 ± 0.45	REACTOME_HIV_INFECTION
LV9	0.31 ± 0.65	−0.17 ± 0.98	0.00 ± 1.24	0.40 ± 1.90	−0.32 ± 1.44	REACTOME_CLASS_I_MHC_MEDIATED_ANTIGEN_ PROCESSING_PRESENTATION
LV10	0.04 ± 0.47	−0.15 ± 0.57	0.11 ± 0.34	0.05 ± 0.31	0.01 ± 0.20	N/A
LV11	−0.41 ± 0.51	0.15 ± 0.93	0.11 ± 0.72	−0.01 ± 0.78	0.37 ± 0.54	N/A
LV12	−0.13 ± 0.61	0.12 ± 0.26	−0.03 ± 0.34	0.08 ± 0.36	0.09 ± 0.26	N/A
LV13^‡^	0.13 ± 0.75	0.35 ± 0.57	0.08 ± 0.56	−0.01 ± 0.51	−1.35 ± 0.27	PID_AR_TF_PATHWAY
LV14^‡^	−0.22 ± 0.74	0.17 ± 0.34	−0.01 ± 0.34	−0.20 ± 0.12	0.12 ± 0.23	N/A
LV15	−0.21 ± 0.79	0.30 ± 0.63	−0.17 ± 0.53	−0.37 ± 0.26	0.20 ± 0.40	REACTOME_TRNA_AMINOACYLATION
LV16**^‡^	−0.38 ± 0.46	0.18 ± 0.36	0.28 ± 0.60	0.34 ± 0.75	−0.21 ± 0.61	N/A
LV17^‡^	0.10 ± 0.89	−0.11 ± 0.71	−0.20 ± 0.68	−0.12 ± 0.59	0.51 ± 0.33	REACTOME_G_ALPHA_I_SIGNALLING_EVENTS
LV18	0.15 ± 0.60	−0.07 ± 0.41	−0.03 ± 0.64	0.02 ± 0.24	−0.10 ± 0.23	N/A
LV19	−0.04 ± 0.42	0.20 ± 0.33	0.01 ± 0.52	−0.30 ± 0.58	−0.43 ± 0.72	N/A
LV20^‡^	0.53 ± 1.93	−0.19 ± 0.57	−0.36 ± 0.49	−0.54 ± 0.28	0.03 ± 0.43	KEGG_CYTOKINE_CYTOKINE_RECEPTOR_INTERACTION
LV21	−0.16 ± 0.59	0.19 ± 0.34	0.03 ± 0.40	−0.03 ± 0.51	−0.13 ± 0.44	REACTOME_GENERIC_TRANSCRIPTION_PATHWAY
LV22*	−0.35 ± 0.69	0.13 ± 0.59	0.33 ± 0.57	0.22 ± 0.78	−0.27 ± 0.36	REACTOME_GENERIC_TRANSCRIPTION_PATHWAY
LV23^‡^	−0.01 ± 1.08	−0.02 ± 0.59	0.27 ± 0.89	0.68 ± 0.17	−0.56 ± 0.47	KEGG_SPLICEOSOME
LV24	0.02 ± 0.33	−0.08 ± 0.71	0.13 ± 0.31	−0.02 ± 0.35	−0.17 ± 0.28	N/A
LV25	−0.11 ± 1.44	0.23 ± 0.77	−0.21 ± 0.72	−0.48 ± 0.63	0.21 ± 0.77	REACTOME_EXTRACELLULAR_MATRIX_ORGANIZATION

Values represent mean within each LV (latent variable) ± standard deviation; YA, young adults; OA, older adults; PD, Parkinson’s disease; RT, resistance training; N/A, PLIER found no annotation using prior knowledge. *P < 0.05 in PD vs. YA; ^†^P < 0.05 in PD vs. both other basal cohorts; **P < 0.05 in YA vs. both other basal cohorts; ^‡^P < 0.05 between PD pre- and post-RT.

### Gene Programs Altered in PD Muscle and Reversed by RT

Table 4 shows 25 LVs identified by PLIER; annotation based on canonical pathways association is provided when available (16 of 25 LVs, 64%) and denoted where not found (N/A). Ten LVs (40%) were significantly different following 16-week RT. Of these, two (LVs 4 and 16) were *both* different at baseline in PD vs. either healthy group *and* significantly reversed following RT. Of the 706 significantly differentially expressed genes in the initial analysis, 38 were in the top 100 of LV4 and 16 were in the top 100 of LV16.

**FIGURE 2:**
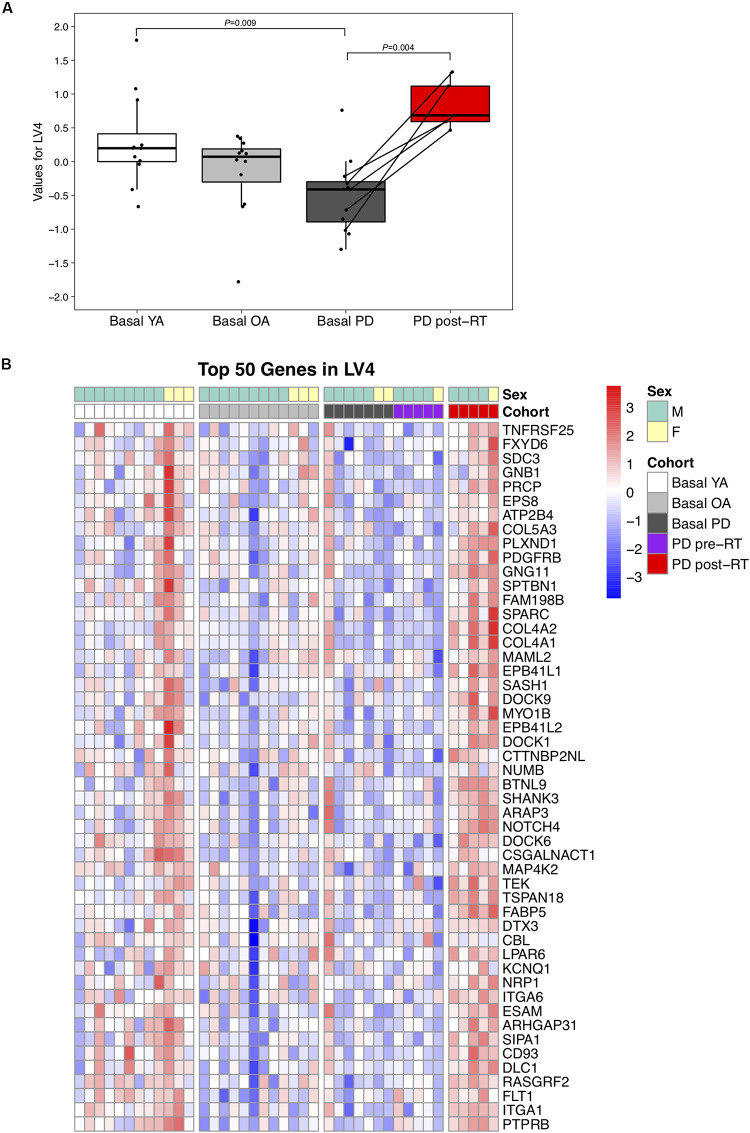
**(A)** Difference in LV (latent variable) 4 across study groups Basal YA (young adults, *n* = 12), Basal OA (older adults, *n* = 12), Basal PD (Parkinson’s disease, *n* = 12), and PD post-RT (rehabilitation training, *n* = 5). Individual points are shown for each subject, and lines represent the change within the five individuals with PD that underwent RT. Among the basal groups, LV4 was significantly lower in PD vs. YA (*P* = 0.009) and increased following RT (*P* = 0.004). **(B)** Heatmap showing normalized expression of the top 50 genes in LV4, illustrating expression profile across study groups. Full gene names are available in Supplementary Data Sheet 3.

LV4 was significantly lower in PD than YA at baseline (*P* = 0.01) and increased following RT (*P* = 0.004, Figure 2A). Normalized expression of the top 50 transcripts in LV4 is presented in Figure 2B. Since the expression pattern was consistent throughout the top 50 genes, manual annotation was performed for the top 100 genes, revealing prominent associations with neuromuscular adaptations to exercise, including neural development (SPTBN1, DOCK6, and DOCK9), muscle development (PDGFRB and COL5A3), and neuroprotective effects (RASGRF2, AGAP2, and ARRB1), as well as skeletal muscle structural adaptations, such as muscle regeneration (SPARC, NUMB, and NOTCH1), angiogenesis (NRP1, TEK, and ARAP3), and extracellular matrix remodeling (COL4A2, HYAL1, and BMP1). See Supplementary Data Sheet 3 for full names of the top 100 genes in LV4.

**FIGURE 3:**
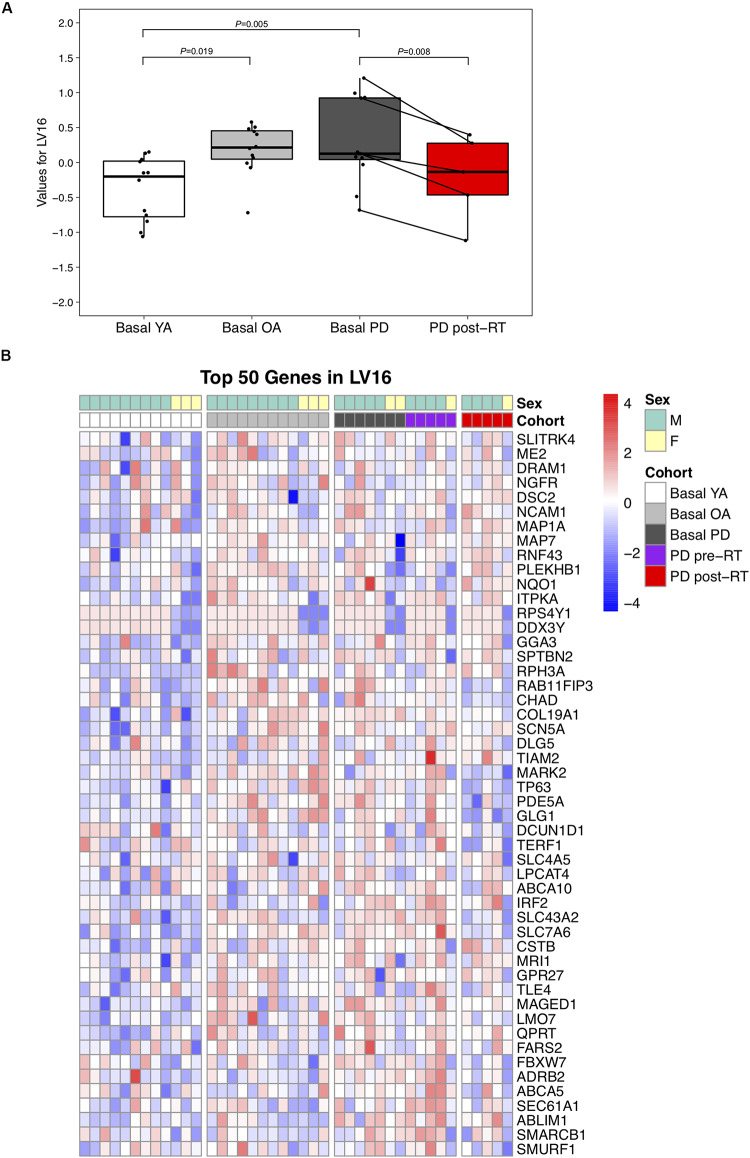
**(A)** Difference in LV (latent variable) 16 across study groups Basal YA (young adults, *n* = 12), Basal OA (older adults, *n* = 12), Basal PD (Parkinson’s disease, *n* = 12), and PD post-RT (rehabilitation training, *n* = 5). Individual points are shown for each subject, and lines represent the change within the five individuals with PD that underwent RT. Among the basal groups, expression of LV16 was significantly higher in both PD (*P* = 0.005) and OA (*P* = 0.019) vs. YA. Expression was reduced following RT (*P* = 0.008). **(B)** Heatmap showing normalized expression of the top 50 genes in LV16, illustrating expression profile across study groups. Full gene names are available in Supplementary Data Sheet 4.

Conversely, LV16 was significantly elevated in PD (*P* = 0.004) and OA (*P* = 0.007) vs. YA at baseline, suggesting a primary effect of age (Figure 3A); RT significantly reduced LV16 (*P* = 0.008). Normalized expression of the top 50 genes in LV16 is presented in Figure 3B. Functional annotation of the top 100 genes in LV16 revealed involvement in age-related muscle dysfunction, e.g., denervation (NCAM, SCN5A, PLEKHB1, and ERBB3), autophagy (DRAM1, RAB23, and TECPR2), apoptosis (DNAJB4, SEC61A1, and GBP2), and inflammation/immunity (TLE4, PDE71, and DUSP10). Supplementary Data Sheet 4 provides full names for the top 100 transcripts in LV16.

## Discussion

This study marks the discovery of two transcriptional programs altered in skeletal muscle by the presence of PD and/or aging and rescued by intensive resistance exercise training. Using the innovative PLIER algorithm to complement a standard differential gene expression workflow, we have revealed coordinated transcriptomic patterns in skeletal muscle associated with PD at baseline and in response to RT. Literature-based interrogation of these transcripts highlights their associations with physiological processes of potential relevance to PD symptomology. In particular, we detected heightened expression of genes associated with muscle regeneration and neural development, and reduced expression of genes associated with apoptosis, autophagy, and immunity. These results provide a potential mechanistic basis for RT-induced improvements in motor and non-motor PD symptoms and direction for future investigations into the impact of intensive exercise rehabilitation on aging and neurodegenerative disease.

### Genes Linked to Exercise-Induced Neuromuscular Adaptations

Utilizing the PLIER framework, we were able to expand greatly upon the single gene-level findings. The finding that a large fraction (16–38%) of the top 100 genes in the LVs of interest were significantly different at the single-transcript level provides a validation of our approach, while further highlighting the utility of network-based analyses to illuminate new relationships among gene sets. At the single-transcript level, we observed patterns that make intuitive sense following RT, such as an increase in expression of genes associated with muscle development and extracellular matrix remodeling [e.g., IGFN (immunoglobulin-like and fibronectin type III domain-containing)1 and a cluster of collagenase enzymes] (Hyldahl et al., 2015; Li et al., 2017) and a decrease in factors that inhibit muscle growth [e.g., MSTN (myostatin), neuronal pentraxin (NPTX)1] (Thalacker-Mercer et al., 2010; Rodriguez et al., 2014; Hooper et al., 2017). Similarities with existing data sets in healthy humans illustrates the important point that skeletal muscle of individuals with PD is responsive to exercise, despite the prominent peripheral manifestation of the disease. Changes in myosin heavy chain isoform expression support our histological evidence of partial type I myofiber grouping reversal with RT (Kelly et al., 2018a): we observed decreased expression of in MYH (myosin heavy chain)1, MYLK (light-chain kinase)2, and others involved in aerobic metabolism, such as PRKAG (protein kinase AMP-activated non-catalytic subunit gamma)3 (Granlund et al., 2011). Application of PLIER allowed us to link other genes to these, amplifying our ability to understand pathways and processes impacted by RT in the PD population.

We previously demonstrated that heightened expression of transcripts involved in neural development was associated with type I grouping in PD skeletal muscle (Lavin et al., 2019), believed to be a result of denervation-reinnervation processes accelerated in PD (Lexell and Downham, 1991; Kelly et al., 2018a). Presently, we detected reversal in direction of expression of a handful of these genes with RT, including transcription factors NFAT (nuclear factor of activated T-cells)5 and ZNF (zinc finger proteins) 689 and 24, the latter of which enables cell cycle progression in early neural development (Khalfallah et al., 2009). Because our small sample size did not mirror the reversal of type I grouping seen in the larger cohort (Kelly et al., 2018a), these findings provide only preliminary insight into the mechanism of type I grouping reversal with exercise. Nevertheless, several genes related to neural development were upregulated as a result of RT, further supporting that neuromuscular communication and likely physical contact may be altered as a result of PD and modulated by exercise. This was a feature of LV4, wherein a number of genes have been linked to in neurite outgrowth, [e.g., SPTBN1 (spectrin beta 1) and DOCK (dedicators of cytokinesis) 6 and 9 (Miyamoto et al., 2007; Kuramoto et al., 2009; Lee et al., 2012)], neural patterning [e.g., MLLT (myeloid/lymphoid or mixed-lineage leukemia; translocated to)4, TSPAN (tetraspanin)18 (Fairchild and Gammill, 2013; Sai et al., 2017)], and axon guidance [e.g., NRP (neuropilin)1, PLXN (plexin)D1, SEMA (semaphorin)5A (Hilario et al., 2009; Pecho-Vrieseling et al., 2009; Korner et al., 2019)]. Larger studies in the future are necessary to determine whether the gene networks we previously identified as correlates of myofiber grouping are impacted during an RT regimen that partially reverses type I grouping or a completely different gene set is involved.

### Genes Linked to Dynamics of Denervation and Neural Apoptosis

Along with a noted increase in expression of genes related to neural functioning and development, RT promoted decreased expression of genes often overexpressed in denervated skeletal muscle. Both SCN5A (voltage-gated sodium channel 5A, known also as Na_v_1.5) and NCAM (neural cell adhesion molecule) were among the top 50 genes grouped into LV16. These factors are often assessed as markers of denervation in adult skeletal muscle (Rowan et al., 2012; Sekiguchi et al., 2012; Hendrickse et al., 2018; Sonjak et al., 2019). The reduction in expression of these transcripts following RT supports our laboratory’s previous findings (Kelly et al., 2018a) and provides direction toward other possible targets for assessment in muscle denervation-reinnervation cycling, including ERBB3 (Erb-B2 receptor tyrosine kinase 3) (Morano et al., 2018) and MAGED1 (melanoma antigen gene family D1) (Magnusson et al., 2005).

Like these denervation markers, expression of genes linked to neural apoptosis was increased with aging and reduced with exercise. Of interest, DNAJ (heat shock family member)B4 enhances apoptosis in neural (Lei et al., 2011) and other (Lin et al., 2010) tissue, while GBP (guanylate binding protein)2 is involved in caspase signaling to promote cell death in neurons (Miao et al., 2017). Additionally, RT reduced expression of SMURF (SMAD-specific E3 ubiquitin ligase)1, which is associated with neuroinflammation and necroptosis (Shao et al., 2018) linking its function to a potential role in neurodegenerative diseases. Conversely, we noted an increase in expression of SCT (secretin), which is reported to cross the blood–brain barrier and may exert neuroprotective effects (Dogrukol-Ak et al., 2004; Martin et al., 2005; Wang et al., 2019). Expression of other neuroprotective genes was upregulated by exercise, including ARRB (arrestin beta)1 (Wang et al., 2014) and AGAP (ArfGAP with GTP-ase domain, ankyrin repeat and pH domain)2. In animals, AGAP2 is sequestered by α-synuclein, a hallmark of PD pathology in other tissues (Atik et al., 2016) leading to death of dopaminergic neurons (Kang et al., 2017). The present findings may suggest that exercise-trained muscle can play an active compensatory role in this balance of neural death and survival in favor of neuroprotection in aging humans. Despite the sample size limitation, the current findings using the novel data integration platform PLIER are nonetheless remarkable and provide a strong basis for future study in larger cohorts.

### Genes Linked to PD Pathology

The skeletal muscle gene signature may provide valuable insight into PD severity, and the plasticity of muscle in response to exercise training may represent an ideal intervention for symptom reversal. ZNF160, recently reported to be a negative circulating biomarker of PD [higher expression associated with a better score on UPDRS, (Santiago and Potashkin, 2017)] was elevated by exercise. Expression of genes linked to abnormal brain and/or neuronal phenotypes in animal models of PD was downregulated by exercise. These include aquaporin (AQP)9 (Stahl et al., 2018), microtubule affinity regulating kinase (MARK)2, and others associated with aggregation of pathological proteins in neurodegenerative conditions [e.g., RANBP (RAN binding protein)10 and ABCA (ATP-binding cassette subfamily A)5 (Kim and Halliday, 2012; Her et al., 2017)]. Like AGAP2 above, SPTBN1 promotes neuronal differentiation but is inhibited by α-synuclein (Lee et al., 2012). Its upregulation at the transcript level in muscle following RT supports a potential compensatory role for skeletal muscle that is enhanced by exercise training. We also detected an increase in FYN (Fyn proto-oncogene), which interacts in microglia with tau (Panicker et al., 2015) a neurofibrillary protein commonly dysregulated in PD (Lei et al., 2010). As this could have potential therapeutic implications, it is necessary to investigate whether skeletal muscle communicates with other relevant tissues, potentially by packaging and releasing factors (e.g., into exosome-like vesicles) that directly interact with α-synuclein and other proteins pathologically altered in PD in order to modulate their impact on functioning. Future research is necessary to determine the communicative role of skeletal muscle in PD before and after an exercise regimen.

Finally, RT induced gene expression of both SIPA1L (signal-induced proliferation associated 1 like)2 and LOXHD (lipoxygenase homology domains)1, genomic variants in which are associated with PD in previous studies (Safaralizadeh et al., 2016; Wang et al., 2016; Gaare et al., 2018; Tao et al., 2019). Presently, there is no available evidence of single-nucleotide polymorphisms in these genes contributing to differential expression in healthy skeletal muscle tissue. Thus, further studies integrating genomic and transcriptomic data are needed in order to elucidate whether increased expression of PD-linked genes with RT represents a direct action of exercise to compensate for PD-associated symptoms at the systemic level. In further support, RASGRF (Ras protein specific guanine nucleotide releasing factor)2, upregulated by RT, is associated with synaptic plasticity (Feig, 2011) and VIPR (vasoactive intestinal peptide receptor)1 may alleviate constipation in PD (Giancola et al., 2017) a distressing non-motor symptom affecting many individuals with PD (Pedrosa et al., 2018).

### Study Limitations

The absence of a control group (e.g., PD cohort remaining sedentary for 16 weeks, or healthy cohort performing RT) is a limitation of this study. We aimed to eliminate the potential influence of this by focusing our attention on LVs that not only changed with RT but changed in the direction of the healthy groups. For example, LV16 was more highly expressed in both older groups (PD and OA) than in young adults, suggesting a primary effect of aging. While many transcripts in this LV were relevant to PD pathologies, future work with a larger sample size is necessary to determine whether these factors are more highly expressed in PD than in OA skeletal muscle at the gene or protein level. Further, our findings are limited to the individuals studied (Hoehn and Yahr stages 2 and 3, mainly male, older adults). Certainly, a larger study sample size would enable detection of smaller fold-changes pre- to post-RT, including a greater number of genes in downstream functional annotation. However, our approach using PLIER allowed us to detect relationships among transcripts differentially expressed at the single gene-level and others that did not reach significance.

Several transcripts of interest identified in this study do not yet have a known biological role (at the protein level) in skeletal muscle. Rather than a limitation, we consider this a benefit of a discovery project, perhaps catalyzing future research. Finally, the skeletal muscle biopsy samples sequenced in this study represent a heterogenous tissue that includes other cell types (Giordani et al., 2019; Rubenstein et al., 2020) meaning that neuronal, glial, or other cell types may have contributed to our detection of neuroactive factors such as SPTBN1 and GBP2. Nevertheless, myofibers make up the vast preponderance of the muscle biopsy specimen; thus, it is most likely that transcripts identified here primarily reflect the transcriptional profile of muscle tissue. The fact that most available evidence regarding the biological roles of these transcripts is drawn from neural cells/tissue highlights the present knowledge gap in potential peripheral roles of these factors in PD. Future work is needed in order to assess whether these transcripts are translated in isolated muscle cells, and subsequently whether they act in an autocrine/paracrine fashion, are degraded, or may be packaged into extracellular vesicles by skeletal muscle.

## Conclusion

This study is the first to examine skeletal muscle gene expression in individuals with Parkinson’s disease following exercise rehabilitation training. In doing so, we identified a robust effect of training, with 706 genes differentially expressed after RT. Application of the PLIER algorithm identified two gene programs that are altered by PD and rescued by RT. The present findings, in combination with our previous work (Kelly et al., 2018a; Lavin et al., 2019) strengthen our understanding of skeletal muscle as a communicative tissue in exercise, aging, and neurodegenerative disease. Further, findings support that, by optimizing muscle health throughout an exercise training regimen, therapeutic effects seen in other tissues and systems may have a mechanistic basis in alterations at the level of the skeletal muscle transcriptome. Future investigations are necessary to determine the influence of exercise training on other levels of phenotype in PD and how skeletal muscle may reflect or orchestrate these changes.

## Data Availability Statement

The datasets generated for this study can be found in the Gene Expression Omnibus GSE140089.

## Ethics Statement

The studies involving human participants were reviewed and approved by University of Alabama Institutional Review Board. The patients/participants provided their written informed consent to participate in this study.

## Author Contributions

KL, SS, and MB contributed to the conception and design of the research. KL, YG, VN, KW, and SW performed the experiments. KL, YG, SS, M-LM, JM, and PK analyzed the data. KL, SS, M-LM, VN, and MB interpreted the results of experiments. KL, YG, and JM prepared the figures. KL drafted the manuscript. KL, YG, SS, M-LM, VN, KW, JM, PK, SW, and MB edited and revised the manuscript and approved the final version of manuscript.

## Conflict of Interest

The authors declare that the research was conducted in the absence of any commercial or financial relationships that could be construed as a potential conflict of interest.

## Footnotes

1 http://www.R-project.org/

## References

[B1] Alkadhi K. A. (2018). Exercise as a positive modulator of brain function. *Mol. Neurobiol.* 55 3112–3130. 10.1007/s12035-017-0516-4 28466271

[B2] Atik A. Stewart T. Zhang J. (2016). Alpha-synuclein as a biomarker for Parkinson’s disease. *Brain Pathol.* 26 410–418. 10.1111/bpa.12370 26940058 PMC6245641

[B3] Azizi S. A. Vendrame M. (2007). Exercise: a workout for neuroregeneration. *Neurosci. Lett.* 418 211–212. 10.1016/j.neulet.2007.03.057 17466455

[B4] Bamman M. M. Petrella J. K. Kim J. S. Mayhew D. L. Cross J. M. (2007). Cluster analysis tests the importance of myogenic gene expression during myofiber hypertrophy in humans. *J. Appl. Physiol.* 102 2232–2239. 10.1152/japplphysiol.00024.2007 17395765

[B5] Benjamini Y. Hochberg Y. (1995). Controlling the false discovery rate: a practical and powerful approach to multiple testing. *J. R. Stat. Soc. B* 57 289–300. 10.1111/j.2517-6161.1995.tb02031.x

[B6] Caviness J. N. Smith B. E. Stevens J. Adler C. H. Caselli R. J. Hentz J. G. (2002). Motor unit number estimates in idiopathic Parkinson’s disease. *Parkinsonism Relat. Disord.* 8 161–164. 10.1016/s1353-8020(01)00007-4 12039425

[B7] Delezie J. Handschin C. (2018). Endocrine crosstalk between skeletal muscle and the brain. *Front. Neurol.* 9:698. 10.3389/fneur.2018.00698 30197620 PMC6117390

[B8] Dobin A. Davis C. A. Schlesinger F. Drenkow J. Zaleski C. Jha S. (2013). STAR: ultrafast universal RNA-seq aligner. *Bioinformatics* 29 15–21. 10.1093/bioinformatics/bts635 23104886 PMC3530905

[B9] Dogrukol-Ak D. Tore F. Tuncel N. (2004). Passage of VIP/PACAP/secretin family across the blood-brain barrier: therapeutic effects. *Curr. Pharm. Des.* 10 1325–1340. 10.2174/1381612043384934 15134484

[B10] Fairchild C. L. Gammill L. S. (2013). Tetraspanin18 is a FoxD3-responsive antagonist of cranial neural crest epithelial-to-mesenchymal transition that maintains cadherin-6B protein. *J. Cell Sci.* 126 1464–1476. 10.1242/jcs.120915 23418345 PMC3644144

[B11] Feig L. A. (2011). Regulation of neuronal function by Ras-GRF exchange factors. *Genes Cancer* 2 306–319. 10.1177/1947601911408077 21779501 PMC3128633

[B12] Ferreira R. M. Alves W. De Lima T. A. Alves T. G. G. Alves Filho P. A. M. Pimentel C. P. (2018). The effect of resistance training on the anxiety symptoms and quality of life in elderly people with Parkinson’s disease: a randomized controlled trial. *Arq. Neuropsiquiatr.* 76 499–506. 10.1590/0004-282x20180071 30231121

[B13] Gaare J. J. Nido G. S. Sztromwasser P. Knappskog P. M. Dahl O. Lund-Johansen M. (2018). Rare genetic variation in mitochondrial pathways influences the risk for Parkinson’s disease. *Mov. Disord.* 33 1591–1600. 10.1002/mds.64 30256453 PMC6282592

[B14] Gentleman R. C. Carey V. J. Bates D. M. Bolstad B. Dettling M. Dudoit S. (2004). Bioconductor: open software development for computational biology and bioinformatics. *Genome Biol.* 5:R80. 10.1186/gb-2004-5-10-r80 15461798 PMC545600

[B15] Giancola F. Torresan F. Repossi R. Bianco F. Latorre R. Ioannou A. (2017). Downregulation of neuronal vasoactive intestinal polypeptide in Parkinson’s disease and chronic constipation. *Neurogastroenterol. Motil.* 29:e12995. 10.1111/nmo.12995 27891695 PMC5393951

[B16] Giordani L. He G. J. Negroni E. Sakai H. Law J. Y. C. Siu M. M. (2019). High-dimensional single-cell cartography reveals novel skeletal muscle-resident cell populations. *Mol. Cell.* 74 609.e6–621.e6. 10.1016/j.molcel.2019.02.026 30922843

[B17] Granlund A. Jensen-Waern M. Essen-Gustavsson B. (2011). The influence of the *PRKAG3* mutation on glycogen, enzyme activities and fibre types in different skeletal muscles of exercise trained pigs. *Acta Vet. Scand.* 53:20. 10.1186/1751-0147-53-20 21435205 PMC3076241

[B18] Hendrickse P. Galinska M. Hodson-Tole E. Degens H. (2018). An evaluation of common markers of muscle denervation in denervated young-adult and old rat gastrocnemius muscle. *Exp. Gerontol.* 106 159–164. 10.1016/j.exger.2018.03.007 29524469

[B19] Her L. S. Mao S. H. Chang C. Y. Cheng P. H. Chang Y. F. Yang H. I. (2017). miR-196a enhances neuronal morphology through suppressing RANBP10 to provide neuroprotection in Huntington’s disease. *Theranostics* 7 2452–2462. 10.7150/thno.18813 28744327 PMC5525749

[B20] Hilario J. D. Rodino-Klapac L. R. Wang C. Beattie C. E. (2009). Semaphorin 5A is a bifunctional axon guidance cue for axial motoneurons in vivo. *Dev. Biol.* 326 190–200. 10.1016/j.ydbio.2008.11.007 19059233

[B21] Hooper A. W. M. Alamilla J. F. Venier R. E. Gillespie D. C. Igdoura S. A. (2017). Neuronal pentraxin 1 depletion delays neurodegeneration and extends life in Sandhoff disease mice. *Hum. Mol. Genet.* 26 661–673. 28007910 10.1093/hmg/ddw422

[B22] Hyldahl R. D. Nelson B. Xin L. Welling T. Groscost L. Hubal M. J. (2015). Extracellular matrix remodeling and its contribution to protective adaptation following lengthening contractions in human muscle. *FASEB J.* 29 2894–2904. 10.1096/fj.14-266668 25808538

[B23] Kang S. S. Zhang Z. Liu X. Manfredsson F. P. He L. Iuvone P. M. (2017). alpha-Synuclein binds and sequesters PIKE-L into Lewy bodies, triggering dopaminergic cell death via AMPK hyperactivation. *Proc. Natl. Acad. Sci. U.S.A.* 114 1183–1188. 10.1073/pnas.1618627114 28096359 PMC5293033

[B24] Kelly N. A. Ford M. P. Standaert D. G. Watts R. L. Bickel C. S. Moellering D. R. (2014). Novel, high-intensity exercise prescription improves muscle mass, mitochondrial function, and physical capacity in individuals with Parkinson’s disease. *J. Appl. Physiol.* 116 582–592. 10.1152/japplphysiol.01277.2013 24408997 PMC4073951

[B25] Kelly N. A. Hammond K. G. Bickel C. S. Windham S. T. Tuggle S. C. Bamman M. M. (2018a). Effects of aging and Parkinson’s disease on motor unit remodeling: influence of resistance exercise training. *J. Appl. Physiol.* 124 888–898. 10.1152/japplphysiol.00563.2017 29357501 PMC5972459

[B26] Kelly N. A. Hammond K. G. Stec M. J. Bickel C. S. Tuggle S. C. Bamman M. M. (2018b). Quantification and characterization of grouped type I myofibers in human aging. *Muscle Nerve* 57 E52–E59. 10.1002/mus.25711 28561923 PMC5711619

[B27] Kelly N. A. Wood K. H. Allendorfer J. B. Ford M. P. Bickel C. S. Marstrander J. (2017). High-intensity exercise acutely increases substantia nigra and prefrontal brain activity in Parkinson’s disease. *Med. Sci. Monit.* 23 6064–6071. 10.12659/msm.906179 29273705 PMC5747933

[B28] Khalfallah O. Ravassard P. Lagache C. S. Fligny C. Serre A. Bayard E. (2009). Zinc finger protein 191 (ZNF191/Zfp191) is necessary to maintain neural cells as cycling progenitors. *Stem Cells* 27 1643–1653. 10.1002/stem.88 19544452

[B29] Kim W. S. Halliday G. M. (2012). Changes in sphingomyelin level affect alpha-synuclein and ABCA5 expression. *J. Parkinsons Dis.* 2 41–46. 10.3233/jpd-2012-11059 23939407

[B30] Korner S. Thau-Habermann N. Kefalakes E. Bursch F. Petri S. (2019). Expression of the axon-guidance protein receptor Neuropilin 1 is increased in the spinal cord and decreased in muscle of a mouse model of amyotrophic lateral sclerosis. *Eur. J. Neurosci.* 49 1529–1543. 10.1111/ejn.14326 30589468

[B31] Kosek D. J. Kim J. S. Petrella J. K. Cross J. M. Bamman M. M. (2006). Efficacy of 3 days/wk resistance training on myofiber hypertrophy and myogenic mechanisms in young vs. older adults. *J. Appl. Physiol.* 101 531–544. 10.1152/japplphysiol.01474.2005 16614355

[B32] Kuramoto K. Negishi M. Katoh H. (2009). Regulation of dendrite growth by the Cdc42 activator Zizimin1/Dock9 in hippocampal neurons. *J. Neurosci. Res.* 87 1794–1805. 10.1002/jnr.21997 19156867

[B33] Lavin K. M. Sealfon S. C. Mcdonald M. N. Roberts B. M. Wilk K. Nair V. D. (2019). Skeletal muscle transcriptional networks linked to type I myofiber grouping in Parkinson’s disease. *J. Appl. Physiol.* 128 229–240. 10.1152/japplphysiol.00702.2019 31829804 PMC7052589

[B34] Lee H. J. Lee K. Im H. (2012). alpha-Synuclein modulates neurite outgrowth by interacting with SPTBN1. *Biochem. Biophys. Res. Commun.* 424 497–502. 10.1016/j.bbrc.2012.06.143 22771809

[B35] Lei J. X. Cassone C. G. Luebbert C. Liu Q. Y. (2011). A novel neuron-enriched protein SDIM1 is down regulated in Alzheimer’s brains and attenuates cell death induced by DNAJB4 over-expression in neuro-progenitor cells. *Mol. Neurodegener.* 6:9. 10.1186/1750-1326-6-9 21255413 PMC3031242

[B36] Lei P. Ayton S. Finkelstein D. I. Adlard P. A. Masters C. L. Bush A. I. (2010). Tau protein: relevance to Parkinson’s disease. *Int. J. Biochem. Cell Biol.* 42 1775–1778. 10.1016/j.biocel.2010.07.016 20678581

[B37] Lexell J. Downham D. Y. (1991). The occurrence of fibre-type grouping in healthy human muscle: a quantitative study of cross-sections of whole vastus lateralis from men between 15 and 83 years. *Acta Neuropathol.* 81 377–381. 10.1007/bf00293457 2028741

[B38] Li X. Baker J. Cracknell T. Haynes A. R. Blanco G. (2017). IGFN1_v1 is required for myoblast fusion and differentiation. *PLoS One* 12:e0180217. 10.1371/journal.pone.0180217 28665998 PMC5493368

[B39] Liao Y. Smyth G. K. Shi W. (2014). featureCounts: an efficient general purpose program for assigning sequence reads to genomic features. *Bioinformatics* 30 923–930. 10.1093/bioinformatics/btt656 24227677

[B40] Lin S. Y. Hsueh C. M. Yu S. L. Su C. C. Shum W. Y. Yeh K. C. (2010). HLJ1 is a novel caspase-3 substrate and its expression enhances UV-induced apoptosis in non-small cell lung carcinoma. *Nucleic Acids Res.* 38 6148–6158. 10.1093/nar/gkq412 20494979 PMC2952861

[B41] Magnusson C. Svensson A. Christerson U. Tagerud S. (2005). Denervation-induced alterations in gene expression in mouse skeletal muscle. *Eur. J. Neurosci.* 21 577–580. 10.1111/j.1460-9568.2005.03855.x 15673457

[B42] Mao W. Zaslavsky E. Hartmann B. M. Sealfon S. C. Chikina M. (2019). Pathway-level information extractor (PLIER) for gene expression data. *Nat. Methods* 16 607–610. 10.1038/s41592-019-0456-1 31249421 PMC7262669

[B43] Martin B. Lopez De Maturana R. Brenneman R. Walent T. Mattson M. P. Maudsley S. (2005). Class II G protein-coupled receptors and their ligands in neuronal function and protection. *Neuromolecular Med.* 7 3–36. 10.1385/nmm:7:1-2:003 16052036 PMC2636744

[B44] Marusiak J. Fisher B. E. Jaskolska A. Slotwinski K. Budrewicz S. Koszewicz M. (2019). Eight weeks of aerobic interval training improves psychomotor function in patients with Parkinson’s disease-randomized controlled trial. *Int. J. Environ. Res. Public Health* 16:880. 10.3390/ijerph16050880 30861998 PMC6427316

[B45] Merritt E. K. Stec M. J. Thalacker-Mercer A. Windham S. T. Cross J. M. Shelley D. P. (2013). Heightened muscle inflammation susceptibility may impair regenerative capacity in aging humans. *J. Appl. Physiol.* 115 937–948. 10.1152/japplphysiol.00019.2013 23681911 PMC3764621

[B46] Miao Q. Ge M. Huang L. (2017). Up-regulation of GBP2 is associated with neuronal apoptosis in rat brain cortex following traumatic brain injury. *Neurochem. Res.* 42 1515–1523. 10.1007/s11064-017-2208-x 28239766

[B47] Miyamoto Y. Yamauchi J. Sanbe A. Tanoue A. (2007). Dock6, a Dock-C subfamily guanine nucleotide exchanger, has the dual specificity for Rac1 and Cdc42 and regulates neurite outgrowth. *Exp. Cell Res.* 313 791–804. 10.1016/j.yexcr.2006.11.017 17196961

[B48] Morano M. Ronchi G. Nicolo V. Fornasari B. E. Crosio A. Perroteau I. (2018). Modulation of the Neuregulin 1/ErbB system after skeletal muscle denervation and reinnervation. *Sci. Rep.* 8:5047. 10.1038/s41598-018-23454-8 29568012 PMC5864756

[B49] Panicker N. Saminathan H. Jin H. Neal M. Harischandra D. S. Gordon R. (2015). Fyn kinase regulates microglial neuroinflammatory responses in cell culture and animal models of Parkinson’s disease. *J. Neurosci.* 35 10058–10077. 10.1523/jneurosci.0302-15.2015 26157004 PMC4495236

[B50] Pecho-Vrieseling E. Sigrist M. Yoshida Y. Jessell T. M. Arber S. (2009). Specificity of sensory-motor connections encoded by Sema3e-Plxnd1 recognition. *Nature* 459 842–846. 10.1038/nature08000 19421194 PMC2847258

[B51] Pedersen B. K. (2019). Physical activity and muscle-brain crosstalk. *Nat. Rev. Endocrinol.* 15 383–392. 10.1038/s41574-019-0174-x 30837717

[B52] Pedrosa A. J. Timmermann L. Pedrosa D. J. (2018). Management of constipation in patients with Parkinson’s disease. *NPJ Parkinsons Dis.* 4:6. 10.1038/s41531-018-0042-8 29560414 PMC5856748

[B53] Petrella J. K. Kim J. S. Tuggle S. C. Bamman M. M. (2007). Contributions of force and velocity to improved power with progressive resistance training in young and older adults. *Eur. J. Appl. Physiol.* 99 343–351. 10.1007/s00421-006-0353-z 17165058

[B54] Petrella J. K. Kim J. S. Tuggle S. C. Hall S. R. Bamman M. M. (2005). Age differences in knee extension power, contractile velocity, and fatigability. *J. Appl. Physiol.* 98 211–220. 10.1152/japplphysiol.00294.2004 15347625

[B55] Piasecki M. Ireland A. Stashuk D. Hamilton-Wright A. Jones D. A. Mcphee J. S. (2016). Age-related neuromuscular changes affecting human vastus lateralis. *J. Physiol.* 594 4525–4536. 10.1113/jp271087 26486316 PMC4983624

[B56] Ritchie M. E. Phipson B. Wu D. Hu Y. Law C. W. Shi W. (2015). limma powers differential expression analyses for RNA-sequencing and microarray studies. *Nucleic Acids Res.* 43:e47. 10.1093/nar/gkv007 25605792 PMC4402510

[B57] Roberts B. M. Lavin K. M. Many G. M. Thalacker-Mercer A. Merritt E. K. Bickel C. S. (2018). Human neuromuscular aging: sex differences revealed at the myocellular level. *Exp. Gerontol.* 106 116–124. 10.1016/j.exger.2018.02.023 29481967 PMC6031257

[B58] Rodriguez J. Vernus B. Chelh I. Cassar-Malek I. Gabillard J. C. Hadj Sassi A. (2014). Myostatin and the skeletal muscle atrophy and hypertrophy signaling pathways. *Cell Mol. Life. Sci* 71 4361–4371. 10.1007/s00018-014-1689-x 25080109 PMC11113773

[B59] Rowan S. L. Rygiel K. Purves-Smith F. M. Solbak N. M. Turnbull D. M. Hepple R. T. (2012). Denervation causes fiber atrophy and myosin heavy chain co-expression in senescent skeletal muscle. *PLoS One* 7:e29082. 10.1371/journal.pone.0029082 22235261 PMC3250397

[B60] Rubenstein A. B. Smith G. R. Raue U. Begue G. Minchev K. Ruf-Zamojski F. (2020). Single-cell transcriptional profiles in human skeletal muscle. *Sci. Rep.* 10:229. 10.1038/s41598-019-57110-6 PMC6959232 31937892

[B61] Safaralizadeh T. Jamshidi J. Esmaili Shandiz E. Movafagh A. Fazeli A. Emamalizadeh B. (2016). SIPA1L2, MIR4697, GCH1 and VPS13C loci and risk of Parkinson’s diseases in Iranian population: a case-control study. *J. Neurol. Sci.* 369 1–4. 10.1016/j.jns.2016.08.001 27653855

[B62] Sai K. Wang S. Kaito A. Fujiwara T. Maruo T. Itoh Y. (2017). Multiple roles of afadin in the ultrastructural morphogenesis of mouse hippocampal mossy fiber synapses. *J. Comp. Neurol.* 525 2719–2734. 10.1002/cne.24238 28498492

[B63] Santiago J. A. Potashkin J. A. (2017). Evaluation of RNA blood biomarkers in individuals at risk of Parkinson’s disease. *J. Parkinsons Dis.* 7 653–660. 10.3233/jpd-171155 28922168

[B64] Sekiguchi K. Kanda F. Mitsui S. Kohara N. Chihara K. (2012). Fibrillation potentials of denervated rat skeletal muscle are associated with expression of cardiac-type voltage-gated sodium channel isoform Nav1.5. *Clin. Neurophysiol.* 123 1650–1655. 10.1016/j.clinph.2012.01.002 22336133

[B65] Shao L. Liu X. Zhu S. Liu C. Gao Y. Xu X. (2018). The role of Smurf1 in neuronal necroptosis after lipopolysaccharide-induced neuroinflammation. *Cell Mol. Neurobiol.* 38 809–816. 10.1007/s10571-017-0553-6 28940129 PMC11481904

[B66] Smedley D. Haider S. Durinck S. Pandini L. Provero P. Allen J. (2015). The BioMart community portal: an innovative alternative to large, centralized data repositories. *Nucleic Acids Res.* 43 W589–W598. 10.1093/nar/gkv350 25897122 PMC4489294

[B67] Sonjak V. Jacob K. J. Spendiff S. Vuda M. Perez A. Miguez K. (2019). Reduced mitochondrial content, elevated ros, and modulation by denervation in skeletal muscle of pre-frail/frail elderly women. *J. Gerontol. A Biol. Sci. Med. Sci.* 74 1887–1895. 10.1093/gerona/glz066 30855073

[B68] Stahl K. Rahmani S. Prydz A. Skauli N. Macaulay N. Mylonakou M. N. (2018). Targeted deletion of the aquaglyceroporin AQP9 is protective in a mouse model of Parkinson’s disease. *PLoS One* 13:e0194896. 10.1371/journal.pone.0194896 29566083 PMC5864064

[B69] Stalberg E. Fawcett P. R. (1982). Macro EMG in healthy subjects of different ages. *J. Neurol. Neurosurg. Psychiatry* 45 870–878. 10.1136/jnnp.45.10.870 7143007 PMC491590

[B70] Tao F. Beecham G. W. Rebelo A. P. Svaren J. Blanton S. H. Moran J. J. (2019). Variation in SIPA1L2 is correlated with phenotype modification in Charcot- Marie- Tooth disease type 1A. *Ann. Neurol.* 85 316–330. 10.1002/ana.25426 30706531 PMC7263419

[B71] Thalacker-Mercer A. E. Dell’italia L. J. Cui X. Cross J. M. Bamman M. M. (2010). Differential genomic responses in old vs. young humans despite similar levels of modest muscle damage after resistance loading. *Physiol. Genomics* 40 141–149. 10.1152/physiolgenomics.00151.2009 19903761 PMC2825766

[B72] Tollar J. Nagy F. Kovacs N. Hortobagyi T. (2019). Two-year agility maintenance training slows the progression of parkinsonian symptoms. *Med. Sci. Sports Exerc.* 51 237–245. 10.1249/mss.0000000000001793 30303934

[B73] Voss M. W. Weng T. B. Narayana-Kumanan K. Cole R. C. Wharff C. Reist L. (2019). Acute exercise effects predict training change in cognition and connectivity. *Med. Sci. Sports Exerc.* 52 131–140. 10.1249/mss.0000000000002115 31385912 PMC7753185

[B74] Wang L. Cheng L. Li N. N. Yu W. J. Sun X. Y. Peng R. (2016). Association of four new candidate genetic variants with Parkinson’s disease in a Han Chinese population. *Am. J. Med. Genet. B Neuropsychiatr. Genet.* 171B 342–347. 10.1002/ajmg.b.32410 26678010

[B75] Wang L. Zhang L. Chow B. K. C. (2019). Secretin prevents apoptosis in the developing cerebellum through Bcl-2 and Bcl-xL. *J. Mol. Neurosci.* 68 494–503. 10.1007/s12031-019-01287-y 30874970

[B76] Wang P. Xu T. Y. Wei K. Guan Y. F. Wang X. Xu H. (2014). ARRB1/beta-arrestin-1 mediates neuroprotection through coordination of BECN1-dependent autophagy in cerebral ischemia. *Autophagy* 10 1535–1548. 10.4161/auto.29203 24988431 PMC4206533

